# Associations between left atrial indices and cardiorespiratory and muscular fitness among physically active military personnel

**DOI:** 10.3389/fcvm.2025.1435818

**Published:** 2025-01-31

**Authors:** Yen-Chen Lin, Pang-Yen Liu, Kun-Zhe Tsai, Wei-Chun Huang, Wen-Chung Yu, Xuemei Sui, Carl J. Lavie, Gen-Min Lin

**Affiliations:** ^1^ Division of Cardiology, Department of Internal Medicine, Linkou Chang Gung Memorial Hospital, Taoyuan, Taiwan; ^2^ Graduate Institute of Clinical Medical Sciences, College of Medicine, Chang Gung University, Taoyuan, Taiwan; ^3^ Department of Internal Medicine, Tri-Service General Hospital, National Defense Medical Center, Taipei, Taiwan; ^4^ Department of Medicine, Hualien Armed Forces General Hospital, Hualien City, Taiwan; ^5^ Department of Stomatology of Periodontology, Mackay Memorial Hospital, Taipei, Taiwan; ^6^ Department of Periodontology, School of Dentistry, Tri-Service General Hospital, National Defense Medical Center, Taipei, Taiwan; ^7^ Department of Medicine, Pingtung Christian Hospital, Pingtung City, Pingtung, Taiwan; ^8^ Department of Business Management, National Sun Yat-sen University, Kaohsiung City, Taiwan; ^9^ College of Medicine, National Yang Ming Chiao Tung University, Taipei, Taiwan; ^10^ Division of Cardiology, Department of Internal Medicine, Taipei Veterans General Hospital, Taipei, Taiwan; ^11^ Department of Exercise Science, Arnold School of Public Health, University of South Carolina, Columbia, SC, United States; ^12^ John Ochsner Heart and Vascular Institute, Ochsner Clinical School, The University of Queensland School of Medicine, New Orleans, LA, United States

**Keywords:** left atrial volume index (LAVI), left atrial stiffness index (LASI), left atrial strain, exercise performance, athletes, echocardiography

## Abstract

**Background:**

Left atrial (LA) size and function are linked to exercise intolerance in heart failure, while associations between LA parameters and exercise performance remain unclear in athletes.

**Objectives:**

This study aimed to identify correlations between echocardiographic LA size, pressure, stiffness, and composite indices, and various exercise performance indicators.

**Methods:**

Echocardiographic parameters were obtained from 181 physically active military personnel receiving regular training and tests for a 3-km run and 2 min of push-ups and sit-ups. The top 16% of runners were compared sex-specifically, with the remaining 84% as controls to identify LA discriminators for running capacity. LA composite indices were defined as the LA volume index (LAVI) divided by the stiffness index (LASI) or pressure index (mitral E/e′). Spearman correlations were used to identify LA correlates with exercise performance. Generalized linear regressions were used to identify LA predictors of exercise performance with adjustments for potential covariates.

**Results:**

The top 16% of runners vs. controls had a lower LASI based on LA volume changes (LASI_v, 0.196 vs. 0.245, *p* = 0.013) and higher LAVI-to-LASI_v (12.30 vs. 8.08, *p* = 0.001) and LAVI-to-(mitral E/e′) (4.37 vs. 3.76, *p* = 0.038) ratios. The LAVI-to-LASI_v ratio was the most highly correlated shared LA parameter for running (|rho| = 0.403), push-up (rho = 0.335), and sit-up (rho = 0.352) performance. The LAVI-to-mitral E/e′ ratio was the most highly correlated, independent, and unique LA parameter for running (|rho| = 0.457) performance.

**Conclusions:**

The LAVI-to-LASI_v ratio, comprising LA size and stiffness information, was the best correlate across exercise types, while the LAVI-to-mitral E/e′ ratio, comprising LA size and pressure information, was the best correlate of an endurance exercise, i.e., 3-km running performance.

## Introduction

While sonographic left atrial (LA) parameters are useful in assessing left ventricular (LV) diastolic function and predicting exercise intolerance in heart failure (1), differential sonographic LA indices with unclear implications in athletes vs. untrained controls have been observed. Body surface area (BSA)-indexed maximum LA volume (LAVI) expansion could be a consequence of LV dysfunction, however, LAVI has been shown to positively correlate with cardiorespiratory fitness in a healthy population (2). Athletes are associated with a larger LAVI (3, 4), and a prospective study showed that exercise training increases LAVI (5). Furthermore, lower peak atrial longitudinal strain (PALS) is associated with a higher risk of all-cause death and heart failure hospitalization (6), but both higher and lower PALS have been repeatedly reported in athletes (3). Although a post-training PALS decline is described in female athletes (6), a lower PALS but larger LA reservoir volume, another LA reservoir function parameter defined as the difference between LA maximum and minimum volumes (LAVmax and LAVmin, respectively), is observed in competitive athletes (7). Finally, a higher LA stiffness index (LASI), usually defined as the mitral E-to-e′ ratio divided by PALS is a heart failure risk and symptom predictor (8–10), and a lower LASI has been observed in athletes (7). Nevertheless, a prospective study showed unaltered LASI after 16 weeks of intensive training in female athletes (6).

While we outlined differential sonographic LA indices in athletes vs. untrained controls above, there are questions for clarification. First, whether differential LA features such as a larger LAVI or lower LASI in athletes directly correlate with exercise performance is uncertain because training volume and intensity could be confounders of both LA adaptation and exercise performance. Second, whether a specific LA characteristic has differential significance for performance in different exercises has not been clearly shown. While we consider the ability of the LA to efficiently uphold adequate LV preload in tachycardia-related short diastolic phases to be important for endurance exercise that requires prolonged cardiac output enhancement, one might think good LA compliance to accommodate repeated reposition-related right ventricular output changes may be crucial in sit-ups. Third, whether a composite LA index incorporating LA parameters from a physiological viewpoint outperforms isolated LA features in correlating with exercise performance is unknown. While a small LAVI limits stroke volume enhancement during exercise, high mean LA pressure is associated with exertional dyspnea. Because LA volume and pressure are interconnected for an individual person, we suspect a physiologically meaningful composite LA index incorporating volume and pressure information, the volume-to-pressure ratio, for example, can better demonstrate LA fitness.

To clarify the above questions, we enrolled physically active military personnel undergoing uniform sleep and food schedules and standardized endurance and strength training in this study. We controlled physical training and investigated the relationship between the performance in different exercises, including 3-km field runs and 2-min sit-up and push-up tests, and composite and separate sonographic parameters evaluating LA size, pressure, stiffness, and emptying and reservoir functions.

## Methods

### Study population

Physically active military personnel with uniform physical training programs, sleep and food schedules, and daily food types were included. They underwent regular tests for 3-km field running and 2 min of push-ups and sit-ups (11–13). Despite regular and uniform training, they were not all at the level of competitive athletes. The sex-specific top 16% of runners (one standard deviation) were compared with the remaining 84% of their colleagues, who were considered the controls for clinical, laboratory, and echocardiographic parameters. Estimated maximal oxygen consumption scaled to the body mass (sVO2max) was estimated based on time for the 3-km running test (11, 14, 15).

### Echocardiography

M-mode and two-dimensional transthoracic echocardiography were performed using a Philips iE33 machine equipped with a 1–5 MHz probe by the same well-trained experienced technician. Speckle tracking echocardiography was performed with aCMQ (automated Cardiac Motion Quantification, Philips) software for PALS in the apical four-chamber view. LAVmax and LAVmin were measured at end-systole and at mitral valve closure in the apical four-chamber view (16). Tissue Doppler echocardiography was performed to obtain the lateral mitral annulus e′ velocity. LV mass (LVM) was calculated using end-diastolic linear echocardiographic parameters with the Devereux and Reichek Cube formula. BSA was calculated using the Du Bois formula. BSA-indexed cardiac chamber dimensions and LVM were calculated for data analysis.

### Isolated and composite sonographic LA parameters

(1) Size: LA size indices included LAVI, LA anteroposterior diameter (LAD), and BSA-indexed LAD (LADI).
(2) Pressure: LA pressure was represented by the mitral E-to-e′ ratio, which is proportionate to LA pressure, given there were no significant mitral valve/annulus diseases, significant aortic valve regurgitation, or left bundle branch block in our population (17).
(3) Emptying function: LA total emptying function was evaluated by the LA emptying volume (LAEV) indexed to BSA (LAEVI) and LA emptying fraction (LAEF). LAEV was the difference between LAVmax and LAVmin. LAEF was defined as the LAEV-to-LAVmax ratio.
(4) Reservoir function: LA reservoir function was evaluated by one-dimensional PALS and volume-based LA reservoir volume fraction (LARVF). LARVF was defined as the LAEV-to-LAVmin ratio.
(5) Stiffness: LASI based on myocardial deformation (LASI_m) was defined as the mitral E/e′-to-PALS ratio (7). LASI based on volume changes (LASI_v) was defined as the mitral E/e′-to-LARVF ratio. LASIes denotes LASI_m and LASI_v together.
(6) Composite indices: the LAVI-to-E/e′ ratio represented the LA volume-to-pressure index. The LAVI-to-LASIes ratios were LA volume-to-stiffness indices.

### Statistical analysis

Analyses were performed using R language in RStudio. The normal distribution of the continuous variables was double-checked using the Shapiro–Wilk and Anderson–Darling normality tests. For a specific continuous variable, data were presented as mean ± standard deviation if a normal distribution was confirmed in both groups and as median and interquartile range [q1, q3] otherwise. Categorical variables were presented as numbers and percentages. Comparisons between two independent groups were made with Welch's *t*-test if normally distributed and the Mann–Whitney *U*-test otherwise. Spearman correlation was used to determine the direction and strength of the monotonic relationship between two independent variables. Distance correlation was used to determine the strength of the association, which was not necessarily linear, between two independent variables. Variables with a significant correlation with exercise performance were further checked with partial correlation to identify independent variables. The partial Spearman correlation was conducted using the ppcor package and partial distance correlation with the energy package (18, 19). Robust generalized linear regression for non-Gaussian distributed continuous variables was constructed using robustbase::glmrob (gamma family). Generalized linear regression (quasi-Poisson family) was conducted to count data such as the number of push-ups and sit-ups. Collinear predictor variables for regression were detected and avoided to keep the Variance Inflation Factor <10, in general, using the usdm package. Statistical significance was defined as a *p*-value <0.05.

## Results

A total of 181, including 159 male and 22 female, physically active military personnel were enrolled. LAVmax and LAVmin were randomly acquired in 96 participants, and PALS was randomly obtained in 124 participants. The sex-specific top 16% of participants, according to their 3-km running performance, were compared with the remaining 84% of their colleagues as controls. The physical and clinical characteristics of the top runners and controls are shown in Table 1. The top runners were younger and had lower waist circumference, waist-to-height ratio (WHtR), and resting pulse rate. The top runners had a higher estimated sVO2max and better performance in push-ups and sit-ups. The echocardiographic variables of both groups are listed in Table 2. The top runners had larger BSA-indexed LV end-diastolic diameter (LVEDDI), LAVI/(E/e′) and LAVI/LASIes, higher mitral E and e′ velocities, and lower LASIes.

**Table 1:** Clinical characteristics and each exercise performance of the top 16% of runners and the controls.

	Top runners (*N* = 30)	Controls (*N* = 151)	*p*-value
Clinical characteristics			
Age (years)	24 (19.5–29)	26 (22–32)	0.036
Males [*n* (%)]	26 (87)	133 (88)	
Height (cm)	171 ± 7.19	171 ± 7.22	0.904
Weight (kg)	68.4 (63.8–74.4)	71.2 (65.0–80.4)	0.156
Body mass index (kg/cm^2^)	23.2 (21.5–26.2)	24.8 (22.6–26.6)	0.083
Body surface area (cm^2^)	1.81 (1.72–1.87)	1.83 (1.74–1.96)	0.345
Waist circumference (cm)	78.2 ± 7.48	82.9 ± 9.17	0.004
Waist-to-height ratio	0.458 ± 0.039	0.486 ± 0.048	0.001
Pulse rate (beats/min)	69 (64–72)	75 (67.5–82)	0.006
Systolic blood pressure (mm Hg)	119 ± 9.28	120 ± 11.8	0.478
Diastolic blood pressure (mm Hg)	71.9 ± 7.01	72.6 ± 10.0	0.617
Current tobacco smoking [*n* (%)]	9 (30)	67 (44)	
Exercise performance			
Estimated sVO2max (L/min)	38.0 (36.8–38.4)	32.2 (28.7–34.0)	<0.001
3-km running time (s)	755 (745–785)	905 (860–995)	<0.001
2-min push-ups (*n*)	50.5 (45–60)	42 (40–50)	<0.001
2-min sit-ups (*n*)	48 (45–50)	42 (40–45)	<0.001

sVO2max = maximal oxygen consumption, scaled to the body mass.

**Table 2:** M-mode, two-dimensional, pulsed wave and tissue Doppler, and speckle tracking echocardiographic parameters of the top 16% of runners and the controls.

	Top runners (*N* = 30)	Controls (*N* = 151)	*p*-value
M-mode and two-dimensional echocardiography			
IVS (mm)	9 (8–9)	9 (8–10)	0.252
IVS, BSA-indexed (mm/m^2^)	4.9 ± 0.546	4.94 ± 0.485	0.773
LVPW (mm)	9 (8–9)	9 (8–9)	0.722
LVPW, BSA-indexed (mm/m^2^)	4.76 ± 0.461	4.88 ± 0.445	0.184
LVEDD (mm)	50.5 (47.2–53)	49 (46–51)	0.064
LVEDDI (mm/m^2^)	27.6 ± 2.22	26.5 ± 2.29	0.019
LV mass, BSA-indexed (g/m^2^)	85.4 ± 15.0	81.6 ± 15.1	0.206
LV ejection fraction (%)	64.5 (61–68)	64 (61–66.5)	0.767
LAD (mm)	33 (30.2–35)	33 (31–35)	0.498
LADI (mm/m^2^)	18.0 (16.9–19.1)	17.8 (16.9–19.1)	0.904
SoV (mm)	28.5 (27–30.8)	29 (27–31)	0.378
SoV, BSA-indexed (mm/m^2^)	15.9 ± 1.52	15.9 ± 1.43	0.943
AVO (mm)	20 (18.2–21)	20 (18.5–21)	0.431
AVO, BSA-indexed (mm/m^2^)	11.1 ± 0.834	10.8 ± 0.995	0.096
RVD, PLAX (mm)	24.8 ± 4.33	25.3 ± 4.02	0.913
RVD, BSA-indexed, PLAX (mm/m^2^)	13.7 ± 2.26	13.8 ± 2.09	0.870
Pulsed wave and tissue Doppler echocardiography			
Mitral E velocity (cm/s)	94.5 ± 12.3	88.1 ± 16.7	0.018
Mitral A velocity (cm/s)	50.6 (45.3–59.5)	51.6 (45.7–59.2)	0.631
Mitral E/A ratio	1.77 (1.70–2.02)	1.7 (1.41–2.00)	0.094
Mitral annulus e′ velocity (cm/s)	19.0 (17.1–21.4)	17.2 (14.2–19.3)	<0.001
Mitral E/e′ ratio	4.9 (4.38–5.28)	5.17 (4.57–5.98)	0.064
Mitral annulus a′ velocity (cm/s)	8.78 (7.38–10.1)	9.26 (8.06–10.7)	0.201
Speckle tracking echocardiography			
LAVI (ml/m^2^)	19.9 (17.9–25.6)	20.2 (16.6–23.8)	0.528
LAEVI (ml/m^2^)	13.0 (9.4–17.2)	12.1 (7.1–15.3)	0.181
LAEF (%)	71.8 (60.3–75.0)	63.4 (51.0–72.3)	0.050
PALS (%)	35 (31.3–40.9)	34.4 (28.8–42.7)	0.598
LARVF (%)	2.55 (1.52–2.98)	1.72 (1.04–2.6)	0.055
LASI_v (ml^−1^)	0.196 (0.139–0.316)	0.245 (0.185–0.444)	0.013
LASI_m (%^−1^)	0.13 (0.116–0.147)	0.151 (0.126–0.183)	0.021
LAVI-to-LASI_m ratio	164 (135–214)	127 (100–170)	0.024
LAVI-to-LASI_v ratio	12.3 (10.4–18.7)	8.08 (5.26–10.7)	0.001
LAVI-to-(mitral E/e′) ratio	4.37 (3.58–6.10)	3.76 (2.95–4.82)	0.038

AVO, aortic cusp separation; BSA, body surface area; IVS, interventricular septum thickness; LAD, left atrial diameter; LADI, left atrial diameter index; LAEF, left atrial emptying fraction; LAEVI, left atrial emptying volume index; LARVF, left atrial reservoir volume fraction; LASI_m, left atrial stiffness index based on myocardial deformation; LASI_v, left atrial stiffness index based on volume changes; LAVI, left atrial volume index; LVEDD, left ventricular end-diastolic diameter; LVPW, left ventricular posterior wall thickness; PALS, peak atrial longitudinal strain; PLAX, parasternal long axis view; RVD, right ventricular diameter; SoV, sinus of Valsalva diameter.

Spearman non-parametric monotonic correlation was applied to find performance correlates, and the results are listed in Table 3. Because sex is a confounder for exercise performance and some clinical and echocardiographic parameters, and most of the participants were men, we did correlation tests for men-only data and included sex in the regression analyses later. Partial Spearman correlation was used to identify independent correlates, among which e′ velocity was a positive performance correlate for each exercise. LVEDDI positively correlated with running and push-ups, whereas LAVI/LASI_v positively correlated with push-up and sit-up ability. LAVI/(E/e′) was a positive, while WHtR was a negative running performance correlate.

**Table 3:** Exercise performance correlates based on Spearman monotonic correlation together with partial Spearman correlation in men.

Performance correlate	Correlation coefficient, rho (95% CI)	*p*-value	Confounding correlates (*p*-value of indirect correlates to predict exercise performance in the partial Spearman correlation)^a^	*N*
Running time (s)				
LAVI/(E/e′)	−0.457 (−0.606 to −0.277)	<0.001	Not found	96
WHtR	0.445 (0.306 to 0.565)	<0.001	Not found	159
e′ velocity	−0.433 (−0.555 to −0.294)	<0.001	Not found	159
LAVI/LASI_v^a^	−0.403 (−0.562 to −0.214)	0.001	LAVI/(E/e′) (0.053)	96
LAVI/LASI_m^a^	−0.401 (−0.561 to −0.212)	0.002	LAVI/(E/e′) (0.544)	96
Waist^a^	0.391 (0.247 to 0.519)	<0.001	WHtR (0.344)	159
BMI^a^	0.354 (0.205 to 0.487)	<0.001	Waist (0.558); WHtR (0.513)	159
LVEDDI	−0.328 ( −0.464 to −0.177)	<0.001	Not found	159
E/A^a^	−0.299 (−0.438 to −0.145)	0.004	e′ velocity (0.548)	159
PR^a^	0.291 (0.137 to 0.431)	0.005	LAVI/(E/e′) (0.132); LAVI (0.051)	159
E/e′^a^	0.273 (0.118 to 0.415)	0.013	e′ velocity (0.997); LAVI/(E/e′) (0.695); WHtR (0.200)	159
AVOI^a^	−0.244 (−0.389 to −0.087)	0.048	LVEDDI (0.247); WHtR (0.222); LAVI/(E/e′) (0.061)	159
Push-up count				
LAVI/LASI_v	0.335 (0.138 to 0.506)	0.029	Not found	96
LVEDDI	0.334 (0.184 to 0.469)	<0.001	Not found	159
LASI_m^a^	−0.296 (−0.453 to −0.121)	0.029	LAVI/LASI_v (0.804)	124
e′ velocity	0.265 (0.110 to 0.408)	0.025	Not found	159
Waist^a^	−0.256 (−0.400 to −0.100)	0.036	LVEDDI (0.200); e′ velocity (0.054); LAVI/LASI_v (0.051)	159
Sit-up count				
LAVI/LASI_v	0.352 (0.157 to 0.520)	0.016	Not found	96
LASI_m^a^	−0.303 (−0.459 to −0.128)	0.022	LAVI/LASI_v (0.773)	124
e′ velocity	0.284 (0.130 to 0.425)	0.010	Not found	159

AVOI, aortic cusp separation index; BMI, body mass index; LASI_m, left atrial stiffness index based on myocardial deformation; LASI_v, left atrial stiffness index based on volume changes; LAVI, left atrial volume index; LVEDDI, left ventricular end-diastolic diameter index; PR, pulse rate; WHtR, waist-to-height ratio.

^a^ Indirect correlates lost their correlation (*p* ≥ 0.05) with exercise performance if each of the confounding correlates is adjusted for.

We next performed distance correlation to detect the correlation strength of any relationship between exercise performance and the characteristics of the male participants, and the results are listed in Table 4. The distance correlation discovered more performance correlates, including all of those in the monotonic correlation. The list of running capacity correlates was the longest, and the strength and rank of the correlates with the different exercises were substantially dissimilar. Partial distance correlation was applied to find independent correlates. LAVI/LASI_v and mitral e′ were directly correlated with performance in all the exercises. WHtR was the most highly correlated anthropometric measurement with running and sit-up performance, while waist circumference was slightly more connected to push-up capacity. LAVI/(E/e′) was a top-ranked and independent running performance correlate. LVEDDI was directly connected to running and push-up performance and correlated with sit-up capacity through LAVI/LASI_v. Mitral E/A was independently correlated with running and push-up performance. LAVI was indirectly linked to running performance via LAVI/LASI_v. Pulse rate was correlated with sit-up capacity through LAVI/LASI_v and with running and push-up capacity through LAVI and all the composite LA indices.

**Table 4:** Exercise performance correlates based on distance correlation together with partial distance correlation in men.

Performance correlate	Correlation coefficient, *r* (95% CI)	*p*-value	Confounding correlates (*p*-value of indirect correlates to predict exercise performance in the partial distance correlation)^a^	*N*
Running time (s)				
WHtR	0.168 (0.013 to 0.316)	<0.001	Not found	159
LAVI/(E/e′)	0.146 (−0.056 to 0.337)	<0.001	Not found	96
e′ velocity	0.145 (−0.011 to 0.294)	<0.001	Not found	159
LAVI/LASI_v	0.126 (−0.076 to 0.318)	<0.001	Not found	96
Waist^a^	0.116 (−0.040 to 0.267)	<0.001	WHtR (1)	159
BMI^a^	0.115 (−0.041 to 0.266)	<0.001	WHtR (0.459)	159
LAVI/LASI_m^a^	0.103 (−0.099 to 0.298)	<0.001	LAVI/(E/e′) (0.134)	96
LVEDDI	0.077 (−0.080 to 0.230)	<0.001	Not found	159
E/A	0.072 (−0.084 to 0.225)	<0.001	Not found	159
LAVI^a^	0.062 (−0.140 to 0.259)	<0.001	LAVI/(E/e′) (0.852); LAVI/LASI_m (0.120)	96
E/e′^a^	0.061 (−0.096 to 0.215)	<0.001	e′ velocity (0.117)	159
PR^a^	0.058 (−0.099 to 0.211)	<0.001	LAVI/(E/e′) (0.080); LAVI (0.062); LAVI/LASI_v (0.059); LAVI/LASI_m (0.053)	159
A velocity	0.049 (−0.107 to 0.203)	<0.001	Not found	159
Age^a^	0.043 (−0.114 to 0.197)	<0.001	LAVI/LASI_v (0.310); LAVI/LASI_m (0.250); LAVI/(E/e′) (0.209); LAVI (0.180); e′ velocity (0.173)	159
Weight^a^	0.041 (−0.115 to 0.196)	<0.001	WhtR (1); Waist (1); BMI (1)	159
AVOI^a^	0.036 (−0.120 to 0.191)	<0.001	WHtR (0.051)	159
E velocity^a^	0.035 (−0.121 to 0.190)	<0.001	LAVI (0.198); LAVI/LASI_v (0.187); LAVI/LASI_m (0.167); LAVI/(E/e′) (0.154); E/A (0.100)	159
LASI_m^a^	0.035 (−0.143 to 0.210)	0.025	LAVI/LASI_v (0.982); LAVI/LASI_m (0.852); e′ (0.251); LAVI/(E/e′) (0.148)	124
DBP^a^	0.033 (−0.123 to 0.188)	0.002	LAVI/(E/e′) (0.516); LAVI/LASI_v (0.482); LAVI (0.463); LAVI/LASI_m (0.460)	159
Push-up count				
LVEDDI	0.088 (−0.069 to 0.240)	<0.001	Not found	159
LAVI/LASI_m	0.068 (−0.135 to 0.264)	<0.001	Not found	96
LAVI/LASI_v	0.066 (−0.136 to 0.263)	<0.001	Not found	96
Waist	0.066 (−0.091 to 0.219)	<0.001	Not found	159
WHtR^a^	0.061 (−0.096 to 0.214)	<0.001	Waist (0.109)	159
LASI_m^a^	0.056 (−0.121 to 0.230)	<0.001	LAVI/LASI_m (0.140); LAVI/LASI_v (0.133)	124
LAVI/(E/e′)^a^	0.052 (−0.150 to 0.250)	0.006	LAVI/LASI_m (0.166)	96
e′ velocity	0.052 (−0.105 to 0.206)	<0.001	Not found	159
AVOI	0.045 (−0.111 to 0.200)	<0.001	Not found	159
BSA^a^	0.043 (−0.113 to 0.197)	<0.001	Waist (0.137)	159
Weight^a^	0.039 (−0.117 to 0.194)	<0.001	Waist (0.871); BSA (0.401); WHtR (0.065)	159
E/A	0.039 (−0.118 to 0.193)	<0.001	Not found	159
E/e′^a^	0.034 (−0.122 to 0.189)	0.002	e′ velocity (0.051)	159
Age	0.032 (−0.124 to 0.186)	0.005	Not found	159
PR^a^	0.026 (−0.130 to 0.181)	0.040	LAVI/(E/e′) (0.472); LAVI (0.433); LAVI/LASI_v (0.425); LAVI/LASI_m (0.417)	159
Sit-up count				
LAVI/LASI_v	0.100 (−0.102 to 0.295)	<0.001	Not found	96
e′ velocity	0.061 (−0.096 to 0.215)	<0.001	Not found	159
LASI_m^a^	0.061 (−0.117 to 0.234)	<0.001	LAVI/LASI_v (0.429)	124
LAVI/LASI_m^a^	0.059 (−0.143 to 0.256)	0.001	LAVI/LASI_v (0.319)	96
Age^a^	0.052 (−0.104 to 0.206)	<0.001	LAVI/LASI_v (0.068)	159
WHtR^a^	0.047 (−0.109 to 0.201)	<0.001	LAVI/LASI_v (0.074)	159
Waist^a^	0.043 (−0.113 to 0.197)	<0.001	LAVI/LASI_v (0.137)	159
LVEDDI^a^	0.037 (−0.119 to 0.191)	<0.001	LAVI/LASI_v (0.094)	159
PR^a^	0.034 (−0.122 to 0.189)	0.002	LAVI/LASI_v (0.085)	159

AVOI, aortic cusp separation index; BMI, body mass index; BSA, body surface area; DBP, diastolic blood pressure; LASI_m, left atrial stiffness index based on myocardial deformation; LASI_v, left atrial stiffness index based on volume changes; LAVI, left atrial volume index; LVEDDI, left ventricular end-diastolic diameter index; PR, pulse rate; WHtR, waist-to-height ratio.

^a^ Indirect correlates lost their correlation (p ≥ 0.05) with exercise performance if each of the confounding correlates is adjusted for.

**Figure 1:**
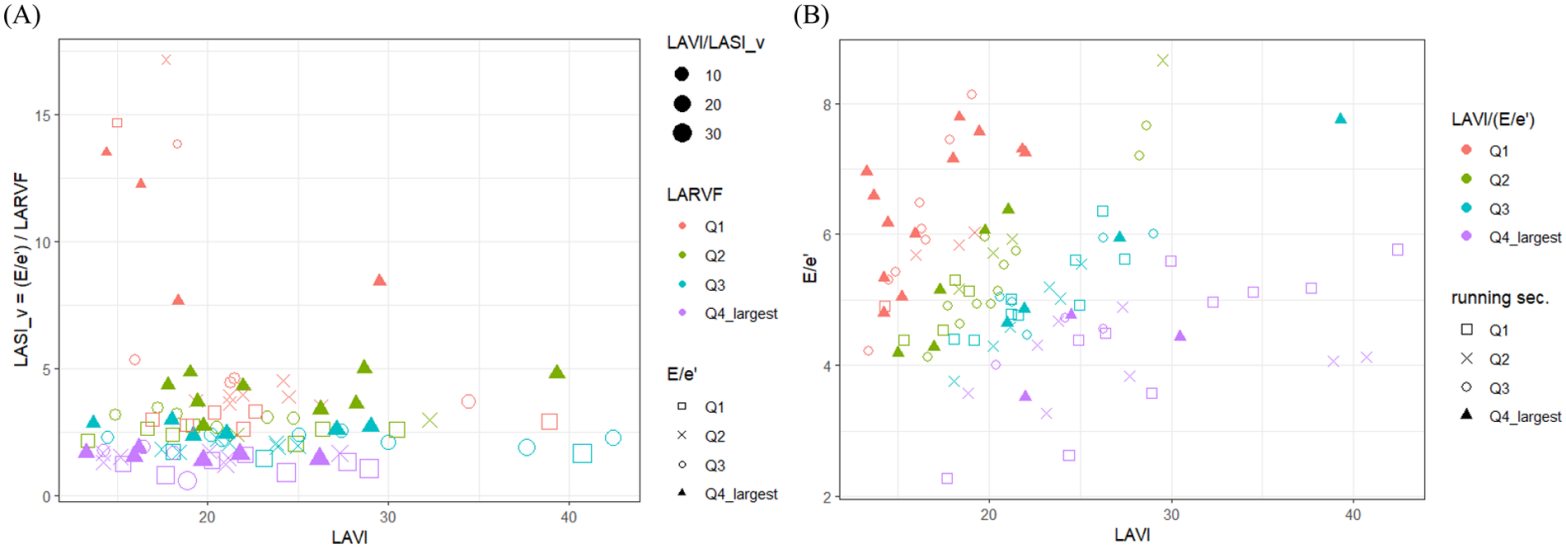
Scatter plots visually depicting (**A**) how variations in LAVI, LARVF, and mitral E/e′ impact the composite LAVI-LASI_v ratio, and (**B**) the relationship between LAVI/(E/e’) and seconds taken to complete a 3-km field run. Q, quartile.

After finding LA features that were differentially correlated with performance in different exercises, we performed regression analyses to clarify the relationship between performance in each exercise and sonographic LA indices, including those evaluating its size (LAD, LADI and LAVI), pressure (E/e′), reservoir ability (PALS and LARVF), total emptying function (LAEVI and LAEF), stiffness (LASIes), and physiologically meaningful composite indices [LAVI/(E/e′), LAVI/LASIes]. Table 5 shows the *p-*values of LA parameters in predicting performance in each exercise based on generalized linear regression, and the dependency information from the partial distance correlation is labeled. The regression coefficients and standard errors, on the link scale, for the significant LA parameters are presented in Supplementary Table S1. Two simple and two complex models were used for the regression analyses. In the simple models, the significance of performance-predicting LA indices in the men-only model held true in the sex-adjusted model. Of note is that the *p*-value of LASI_m decreased substantially after including women. For the complex models, model_epi controlled clinical data, including age, sex, WHtR, and SBP, while model_dCor adjusted performance correlates, including e′ velocity, LVEDDI, WHtR, and sex. For LA size, LAVI was directly linked to running capacity, while LAD and LADI were not performance predictors. For LA emptying functions, LAEVI no longer predicted running performance in model_dCor, whereas LAEF was not a predictor at all. For LA reservoir functions, LARVF was associated with sit-up capacity, probably through LAVI/LASI_v, and PALS alone did not predict performance. For the LA pressure surrogate, the ability of mitral E/e′ to predict exercise performance in the simple models disappeared after adjusting for e′ velocity (data not shown). For the LA stiffness indices, LASI_v alone was not predictive, while LASI_m lost its connection to running and sit-up performance in model_dCor. Furthermore, the significance of LASI_m in the complex models disappeared without women. For the LA composite indices, LAVI/LASI_v always predicted running and sit-up performance, while LAVI/LASI_m was not a predictor in model_dCor. Figure 1A shows while LAVI and LASI_v interactively shaped LAVI/LASI_v, LARVF surpassed mitral E/e′ in deciding LASI_v in our study population. Finally, LAVI/(E/e′) predicted running performance in all models. Figure 1B depicts the negative correlation between LAVI/(E/e′) and seconds taken to complete a 3-km field run.

**Table 5:** *p*-values of left atrial parameters in predicting each exercise performance based on generalized linear regression.

	Size		Emptying		Pressure		Reservoir		Stiffness		Composite		
	LAD	LADI	LAVI	LAEVI	LAEF	E/e′	PALS	LARVF	LASI_m	LASI_v	LAVI/LASI_m	LAVI/LASI_v	LAVI/(E/e′)
Running time in seconds			indir^a^	uncor^a^		indir^a^			indir^a^		indir^a^	Direct^a^	Direct^a^
crude_men	0.150	0.824	<0.001	0.035	0.505	<0.001	0.593	0.413	0.040	0.680	<0.001	<0.001	<0.001
model_sex	0.124	0.755	<0.001	0.034	0.461	<0.001	0.469	0.354	0.040	0.987	<0.001	<0.001	<0.001
model_epi	0.188	0.635	<0.001	0.011	0.481	0.071	0.888	0.193	0.022	0.795	0.001	<0.001	<0.001
model_dCor	0.139	0.612	0.014	0.053	0.130	0.840	0.947	0.075	0.497	0.626	0.103	0.005	0.013
Push-up count						indir^a^			indir^a^		Direct^a^	Direct^a^	indir^a^
crude_men	0.350	0.148	0.062	0.119	0.481	0.006	0.474	0.534	0.013	0.314	0.017	0.042	0.010
model_sex	0.359	0.142	0.062	0.123	0.527	0.011	0.332	0.460	0.009	0.573	0.013	0.041	0.012
model_epi	0.504	0.074	0.068	0.115	0.658	0.176	0.079	0.427	0.017^b^	0.594	0.002	0.070	0.071
model_dCor	0.524	0.784	0.431	0.256	0.265	0.354	0.076	0.227	0.038^b^	0.338	0.196	0.235	0.462
Sit-ups count				Uncor^a^		Uncor^a^		Uncor^a^	indir^a^		indir^a^	Direct^a^	Uncor^a^
crude_men	0.320	0.997	0.256	0.043	0.129	0.016	0.319	0.040	0.005	0.351	0.003	<0.001	0.003
model_sex	0.282	0.971	0.306	0.050	0.133	0.024	0.265	0.030	0.013	0.538	0.004	<0.001	0.005
model_epi	0.788	0.789	0.383	0.067	0.248	0.138	0.166	0.046	0.032	0.557	0.006	0.001	0.017
model_dCor	0.727	0.514	0.788	0.163	0.068	0.400	0.257	0.017	0.572	0.349	0.432	0.038	0.878

LAD, left atrial diameter; LADI, left atrial diameter index; LAEF, left atrial emptying fraction; LAEVI, left atrial emptying volume index; LARVF, left atrial reservoir volume fraction; LASI_m, left atrial stiffness index based on myocardial deformation; LASI_v, left atrial stiffness index based on volume changes; LAVI, left atrial volume index; PALS, peak atrial longitudinal strain.

Independent parameters: the specific LA parameter of men in crude_men; the specific LA parameter and sex in model_sex; the specific LA parameter, age, sex, WHtR, and SBP in model_epi; the specific LA parameter, e′ velocity, LVEDDI, and WHtR in model_dCor.

^a^ The result of distance and partial distance correlation in Table 4. “Direct” represents an independent correlate while “indir” (indirect) means depending on at least one confounding correlate to be performance correlated. “Uncor” is short for uncorrelation.

^b^ The *p*-value of LASI_m in predicting exercise capacity in model_epi and model_dCor in men is 0.640 and 0.810, respectively.

## Discussion

We identified clinical and echocardiographic indices that correlated with performance in a 3-km field run and 2 min of push-ups and sit-ups in the same group of physically active military personnel receiving uniform exercise training, sleep and food schedules, and diet. By comparing the performance in different exercises in the same population, we controlled for interpersonal diversity to identify sonographic indices that were differential and significant for running, push-up, and sit-up capacity. In addition, body training might have confounding effects on both exercise performance and sonographic parameters, and sleep and food schedules and diet interfere with exercise capacity (20, 21). After controlling for the above-mentioned confounding factors, we showed that LAVI/LASI_v was the most prominent common LA correlate with running, sit-up, and probably push-up performance, while LAVI/(E/e′) was the strongest, most direct, and relatively specific LA correlate with running capacity. We concluded that, compared to separate LA variables, composite LA indices properly incorporating LA volume with pressure or stiffness information are more highly correlated with overall exercise performance.

### LAVI but not LAD nor LADI is correlated with endurance

By controlling physical training in military personnel, we showed that LAVI was positively correlated with and predictive of running performance. An increased LAVI is among the presentations of an athlete's heart (22). In a prospective study of top-level female athletes, LAVI increased without filling pressure changes after a 16-week intensive training program (6). Another study showed that 10 months of high-intensity exercise led to comparable rises in LAVmax, LV end-diastolic volume, and VO2max (5). A larger LAVI represents higher LA potential for efficiently boosting total emptying volume in tachycardia-related short diastolic phases to support cardiac output enhancement during endurance exercise. In contrast to LAVI, LAD and LADI did not reflect LA volume and failed to predict field running performance.

### LAVI outperforms LAEVI in predicting running performance for hemodynamic reasons

Resting total LA emptying parameters were not as predictive of running performance as LAVI. LAEF represented LA emptying volume scaled to the LA but not body size and was unconnected to exercise capacity. LAEVI lost its power to predict sit-up performance in our complex models and, to our surprise, running performance in model_dCor. A real-time magnetic resonance imaging-based study shows that during supine bicycle exercise, LAEVI enhancement results from reduced LAVmin with unchanged LAVI in both athletes and non-athletes. Exercise induces comparable LAEF increase and BSA-indexed LAVmin decrease in athletes and non-athletes, and a larger LAVI was the main determinant of a larger exercise LAEVI (23). Similarly, our finding implied that resting LAVI vs. LAEVI is more representative of exercise LAEVI. Hence, the LAVI was used to represent the stroke volume reserve scaled to the body size in our composite LA indices.

### Proper combination of LA hemodynamic parameters helps in performance prediction

LA reservoir indices and their derived LA stiffness variables behaved quite differently with regard to their correlation with and prediction of exercise performance. PALS and LARVF did not represent absolute LA reservoir volume; however, PALS was performance-unrelated, whereas LARVF predicted sit-up capacity. The reason behind this phenomenon was unclear, but there was a link between sit-ups and LARVF. Sit-ups involve reposition-related cyclic volumetric changes in venous return and, thus right ventricular output to the LA, while LARVF represents LA capacity for global volume expansion. Interestingly, PALS-based LASI_m was significantly correlated with performance in all the exercises except in model_dCor, while to our surprise, LARVF-based LASI_v was not predictive of exercise performance. These findings might be explained by the fact that PALS and mitral E and e′ wave velocities, unlike LARVF, are all one-dimensional measurements in the longitudinal direction in the four-chamber view, and therefore only LASI_m truly represented LA stiffness. These observations suggest that properly combining isolated LA hemodynamic parameters helped in predicting exercise performance.

### LAVI-based composite hemodynamic indices are better performance correlators

Unsurprisingly, compared to individual LA parameters, we showed that physiologically meaningful composite LA variables combining LA volume with pressure or stiffness information were more highly correlated with exercise performance. While a larger LAVI signified a better ability to increase exercise LV preload without providing information on associated costs, larger LAVI/(E/e′) and LAVI/LASI_m ratios represented a higher LA-contributed reserve at a unit expense of LA pressure and stiffness, respectively. The reason behind the dramatically improved performance correlation from LASI_v to LAVI/LASI_v not determined, but perhaps because the latter could be re-formulated as {[(LAVmax/LAVmin) * (LAVmax − LAVmin)]/BSA}/(E/e′). While the {numerator} described detailed LA expansive abilities scaled to the body size, the denominator again provided the accompanied expense of LA pressure. The reasons why LAVI/(E/e′) and LAVI/LASIes were differentially correlated with the performance of different exercises were uncertain, and the need for endurance and cardiac cycle-unrelated variations of LA preload and afterload might play a role. The ability of the LA to boost exercise LAEVI without significant LA and pulmonary venous pressure rises is crucial for 3-km field running, which requires endurance and does not involve significant cardiac cycle-unrelated variations in LV preload and afterload. In contrast, the ability of the LA to accommodate reposition-related variations in venous return and peripheral resistance might play a role in sit-up and push-up performance, respectively.

### LAVI-based hemodynamic indices connect resting heart rate with exercise performance

LAVI-based LA hemodynamic indices provide more comprehensive information to assess LA fit, and we showed that resting heart rate, lower in the top runners, was negatively correlated with exercise performance through these composite parameters. Resting heart rate is usually reported to be lower in athletes vs. controls and in athletes with vs. those without a dilated LA (24–26). A larger LAVI potentially ensures larger stroke volume and a lower heart rate is enough to maintain adequate resting cardiac output. Nevertheless, in a trial of supine bicycle exercise, athletes had a trend of lower resting but higher exercise heart rates (23). With a larger LAVI and normal or supernormal exercise heart rate response, athletes increase cardiac outputs to support musculoskeletal systems and have extraordinary exercise performance.

### Major common non-LA performance correlators

Exercise performance is undoubtedly influenced by multiple non-LA factors. Our distance correlation analyses indicated that mitral e′ velocity, LVEDDI, and WHtR were dominant and shared non-LA parameters correlating with all exercise performance. Mitral e′ velocity is roughly inversely proportionate to the LV relaxation time constant, and is determined by LV relaxation, restoring forces, and filling pressure in the general population (1, 17). LV diastolic function, crucial for athletes, positively correlated with exercise performance in this study. An appropriately larger LVEDDI, according to the Frank–Starling law, represents the capacity of the LV to enhance stroke volume while lessening forceful contraction, which is highly energy-consuming. WHtR is a surrogate measurement of body fat percentage, which in contrast to muscles, increases weight burden without improving exercise ability. People with a high WHtR also have higher cardiometabolic and mortality risks (27). Compared to BMI and body weight, WHtR was negatively correlated with all exercise performance in our study. Therefore, we included mitral e′ velocity to represent LV diastolic function, LVEDDI to represent stroke volume reserve, and WHtR to represent fat burden and cardiometabolic risk in our generalized linear regression analyses.

### Potential application of composite LA hemodynamic indices in practice

While we proved that properly incorporating interconnected LA indices from an exercise type-specific physiologic viewpoint benefited fitness assessments, one might try to improve exercise capacities or adjust training programs based on this knowledge. For example, because endurance training is most known to be associated with a larger LAVI, which enhances stroke volume, strength athletes might improve performance by including suitable endurance activities in their daily training. Furthermore, because pathological conditions and probably also inappropriately violent training increase the risk of stretch-associated atrial fibrosis, which increases LA stiffness, periodical assessments of LA health with LAVI/LASIes might contribute to athlete-focused echocardiographic evaluations and help to tailor training programs individually (28).

## Study limitations

First, we did not evaluate LA conduit and active pumping functions, nor did we separate PALS into LA conduit and contraction strains. Total LA conduit volume is the difference between LV stroke volume and total LA reservoir volume; however, we were unable to obtain LV stroke volume for its calculation. Second, we used the apical four-chamber but not the two-chamber view to evaluate PALS and LA volumes. Third, we combined men and women to compare sex-specific top runners with controls. For example, LASI_m was not a significant push-up performance predictor in model_epi and model_dCor if women were excluded. However, removing female data did not change the predictive significance of the other LA parameter-exercise performance combinations. Fourth, we could only evaluate resting echocardiographic parameters, which might substantially change during exercise. Fifth, exercise performance is determined not only by cardiac functions and structures but also by musculoskeletal robustness, mental health, and exercise skills. Cardiac fitness might not be the leading factor affecting exercise capacity. Sixth, all echocardiographic parameters were obtained by a well-trained and experienced echocardiographer, but we could not analyze measurement variability because most of the parameters were clearly measured only once in sinus rhythm, and there was no suitable internal control. Finally, physically active military personnel were enrolled to control for confounding and interfering factors, as mentioned above, and the finding might not be extrapolated to a real-world general population.

## Conclusions

Specific LA characteristics play roles of different importance with regard to correlating with and predicting the performance in different exercises. A larger LAVI, which efficiently boosts LV preload in tachycardia-related short diastolic phases to support prolonged stroke volume enhancement during endurance exercise, is not a performance-irrelevant consequence of physical training and is uniquely and positively associated with running performance. Physiologically meaningful composite LA indices combining LAVI with information on LA pressure and stiffness improve the strength and significance of the correlation with and prediction of performance in not only field running but also sit-ups and probably push-ups. Applying composite LA indices from an exercise type-specific physiological viewpoint to routine practice might benefit practical fitness assessments or interventions in military or athletic populations.

### Prospectives

#### Competency in medical knowledge

LA volume and functional indices could be used in conjunction with clinical characteristics and sonographic LV parameters to evaluate potential exercise capacity in physically fit people.

#### Translational outlook

It is sometimes difficult to differentiate physiological adaptation-related larger LA volume from pathological LA dilation. LAVI-to-LASI and LAVI-to-(mitral E/e′) ratios comprising LA volume with concurrent information on LA stiffness and pressure, respectively, could better describe LA fitness. These functional indices have the potential to be extrapolated across the population spectrum from a physiological viewpoint. While we have proved composite LAVI functional indices are useful in physically fit people, whether these indices play roles in exercise tolerance or clinical outcomes in patients with LV diastolic dysfunction, heart failure, or cardiovascular diseases requires additional research.

## Data Availability

The raw data supporting the conclusions of this article will be made available by the authors, without undue reservation.

## References

[1] Nagueh SF Smiseth OA Appleton CP Byrd BF Dokainish H Edvardsen T Recommendations for the evaluation of left ventricular diastolic function by echocardiography: an update from the American society of echocardiography and the European association of cardiovascular imaging. J Am Soc Echocardiogr. (2016) 29(4):277–314. 10.1016/j.echo.2016.01.011 27037982

[2] Letnes JM Nes B Vaardal-Lunde K Slette MB Mølmen-Hansen HE Aspenes ST Left atrial volume, cardiorespiratory fitness, and diastolic function in healthy individuals: the HUNT study, Norway. J Am Heart Assoc. (2020) 9(3):e014682. 10.1161/JAHA.119.014682 31986991 PMC7033857

[3] Cuspidi C Tadic M Sala C Gherbesi E Grassi G Mancia G. Left atrial function in elite athletes: a meta-analysis of two-dimensional speckle tracking echocardiographic studies. Clin Cardiol. (2019) 42(5):579–87. 10.1002/clc.23180 30907013 PMC6523010

[4] Iskandar A Mujtaba MT Thompson PD. Left atrium size in elite athletes. JACC Cardiovasc Imaging. (2015) 8(7):753–62. 10.1016/j.jcmg.2014.12.032 26093921

[5] Opondo MA Aiad N Cain MA Sarma S Howden E Stoller DA Does high-intensity endurance training increase the risk of atrial fibrillation? Circ Arrhythm Electrophysiol. (2018) 11(5):e005598. 10.1161/CIRCEP.117.005598 29748195 PMC5951393

[6] D’Ascenzi F Pelliccia A Natali BM Zacà V Cameli M Alvino F Morphological and functional adaptation of left and right atria induced by training in highly trained female athletes. Circ Cardiovasc Imaging. (2014) 7(2):222–9. 10.1161/CIRCIMAGING.113.001345 24470314

[7] D’Ascenzi F Pelliccia A Natali BM Cameli M Andrei V Incampo E Increased left atrial size is associated with reduced atrial stiffness and preserved reservoir function in athlete’s heart. Int J Cardiovasc Imaging. (2015) 31(4):699–705. 10.1007/s10554-015-0600-7 25627780

[8] Kim D Seo JH Choi KH Lee SH Choi JO Jeon ES Prognostic implications of left atrial stiffness Index in heart failure patients with preserved ejection fraction. Cardiovasc Imaging. (2023) 16(4):435–45. 10.1016/j.jcmg.2022.11.002 36752431

[9] Reddy YNV Obokata M Egbe A Yang JH Pislaru S Lin G Left atrial strain and compliance in the diagnostic evaluation of heart failure with preserved ejection fraction. Eur J Heart Fail. (2019) 21(7):891–900. 10.1002/ejhf.1464 30919562

[10] Singleton MJ Nelson MB Samuel TJ Kitzman DW Brubaker P Haykowsky MJ Left atrial stiffness Index independently predicts exercise intolerance and quality of life in older patients with obese HFpEF. J Card Fail. (2022) 28(4):567–75. 10.1016/j.cardfail.2021.10.010 34774747 PMC9018494

[11] Lin GM Li YH Lee CJ Shiang J-C Lin K-H Chen K-W Rationale and design of the cardiorespiratory fitness and hospitalization events in armed forces study in eastern Taiwan. World J Cardiol. (2016) 8(8):464–71. 10.4330/wjc.v8.i8.464 27621774 PMC4997527

[12] Liu MY Liu PY Tsai KZ Lima JAC Lavie CJ Lin GM. Asian Female athlete’s heart: the CHIEF heart study. Acta Cardiol Sin. (2023) 39(6):888–900. 10.6515/ACS.202311_39(6).20230306F 38022423 PMC10646586

[13] Liu PY Tsai KZ Lima JAC Lavie CJ Lin GM. Athlete’s heart in Asian military males: the CHIEF heart study. Front Cardiovasc Med. (2021) 8. 10.3389/fcvm.2021.725852 34660727 PMC8511640

[14] Lin GM Tsai KZ Sui X Lavie CJ. Estimated power output for a distance run and maximal oxygen uptake in young adults. Front Physiol. (2023) 14:1110802. 10.3389/fphys.2023.1110802 36824464 PMC9941527

[15] Lin GM Tsai KZ Lavie CJ. Waist-to-height ratio for the obesity paradox in heart failure: is it a matter of fitness? Eur Heart J. (2023) 44(35):3386–7. 10.1093/eurheartj/ehad503 37529967

[16] Kou S Caballero L Dulgheru R Voilliot D De Sousa C Kacharava G Echocardiographic reference ranges for normal cardiac chamber size: results from the NORRE study. Eur Heart J Cardiovasc Imaging. (2014) 15(6):680–90. 10.1093/ehjci/jet284 24451180 PMC4402333

[17] Park JH Marwick TH. Use and limitations of E/e’ to assess left ventricular filling pressure by echocardiography. J Cardiovasc Ultrasound. (2011) 19(4):169–73. 10.4250/jcu.2011.19.4.169 22259658 PMC3259539

[18] Székely GJ Rizzo ML. Partial distance correlation with methods for dissimilarities. The Annals of Statistics. (2014) 42(6):2382–412. 10.1214/14-AOS1255

[19] Kim S. Ppcor: an R package for a fast calculation to semi-partial correlation coefficients. Commun Stat Appl Methods. (2015) 22(6):665–74. 10.5351/CSAM.2015.22.6.665 26688802 PMC4681537

[20] Watson AM. Sleep and athletic performance. Curr Sports Med Rep. (2017) 16(6):413. 10.1249/JSR.0000000000000418 29135639

[21] Kerksick CM Arent S Schoenfeld BJ Stout JR Campbell B Wilborn CD International society of sports nutrition position stand: nutrient timing. J Int Soc Sports Nutr. (2017) 14(1):33. 10.1186/s12970-017-0189-4 28919842 PMC5596471

[22] Palermi S Cavarretta E D’Ascenzi F Castelletti S Ricci F Vecchiato M Athlete’s heart: a cardiovascular step-by-step multimodality approach. Rev Cardiovasc Med. (2023) 24(5):151. 10.31083/j.rcm2405151 39076743 PMC11273059

[23] Schnell F Claessen G La Gerche A Claus P Bogaert J Delcroix M Atrial volume and function during exercise in health and disease. J Cardiovasc Magn Reson. (2016) 19(1):104. 10.1186/s12968-017-0416-9 PMC5735907 29254488

[24] Caselli S Di Paolo FM Pisicchio C Pandian NG Pelliccia A. Patterns of left ventricular diastolic function in Olympic athletes. J Am Soc Echocardiogr. (2015) 28(2):236–44. 10.1016/j.echo.2014.09.013 25441331

[25] Trivedi SJ Claessen G Stefani L Flannery MD Brown P Janssens K Differing mechanisms of atrial fibrillation in athletes and non-athletes: alterations in atrial structure and function. Eur Heart J Cardiovasc Imaging. (2020) 21(12):1374–83. 10.1093/ehjci/jeaa183 32757003

[26] Park JH Kim KH Rink L Hornsby K Cho JY Cho G-Y Left atrial enlargement and its association with left atrial strain in university athletes participated in 2015 Gwangju Summer Universiade. Eur Heart J Cardiovasc Imaging. (2020) 21(8):865–72. 10.1093/ehjci/jeaa084 32380526

[27] Savva SC Lamnisos D Kafatos AG. Predicting cardiometabolic risk: waist-to-height ratio or BMI. A meta-analysis. Diabetes Metab Syndr Obes. (2013) 6:403–19. 10.2147/DMSO.S34220 24179379 PMC3810792

[28] Palermi S Serio A Vecchiato M Sirico F Gambardella F Ricci F Potential role of an athlete-focused echocardiogram in sports eligibility. World J Cardiol. (2021) 13(8):271–97. 10.4330/wjc.v13.i8.271 34589165 PMC8436685

