# Supplementary material for: Associations between left atrial indices and cardiorespiratory and muscular fitness among physically active military personnel
Source: Front Cardiovasc Med. 2025 Jan 31;12:1435818. doi: 10.3389/fcvm.2025.1435818 (PMC11825789; doi:10.3389/fcvm.2025.1435818)
Supplement: Supplementary file 1 [file Table1.pdf]

SUPPLEMENTARY TABLE 1 Regression Coefficients and Standard Errors, on the Link Scale, of Left Atrial Parameters with p <0.05 in Predicting Each Exercise Performance Based on Generalized Linear Regression

		Size	Emptying	Pressure	Reservoir	Sfiffness	Composite		
		LAVI	LAEVI	E/e'	LARVF	LASI_m	LAVI/LASI_m	LAVI/LASI_v	LAVI/(E/e')
**(A) running seconds**		indir*	uncor*	indir*		indir*	indir*	DIRECT*	DIRECT*
crude_men	RC	7.099E-06	3.642E-06	-3.129E-05		-4.571E-04	7.372E-07	8.692E-06	3.460E-05
	SE	1.995E-06	1.727E-06	8.247E-06		2.226E-04	1.994E-07	2.207E-06	7.491E-06
model_sex	RC	6.729E-06	3.316E-06	-2.713E-05		-3.751E-04	6.428E-07	7.754E-06	3.340E-05
	SE	1.874E-06	1.566E-06	7.522E-06		1.822E-04	1.811E-07	1.980E-06	7.058E-06
model_epi	RC	6.246E-06	3.393E-06			-3.731E-04	5.897E-07	7.365E-06	2.835E-05
	SE	1.686E-06	1.336E-06			1.631E-04	1.710E-07	1.805E-06	6.802E-06
model_dCor	RC	4.114E-06						5.148E-06	2.050E-05
	SE	1.678E-06						1.839E-06	8.289E-06
**(B) push-ups counts**				indir*		indir*	DIRECT*	DIRECT*	indir*
crude_men	RC			-4.919E-02		-1.211E+00	9.778E-04	8.480E-03	3.986E-02
	SE			1.768E-02		4.794E-01	4.015E-04	4.402E-03	1.521E-02
model_sex	RC			-4.449E-02		-1.172E+00	9.793E-04	8.294E-03	3.853E-02
	SE			1.720E-02		4.410E-01	3.894E-04	4.239E-03	1.509E-02
model_epi	RC					-1.063E+00	8.690E-04	7.129E-03	2.776E-02
	SE					4.382E-01	3.793E-04	4.159E-03	1.520E-02
model_dCor	RC					-9.686E-01			
	SE					4.634E-01			
**(C) sit-ups counts**			Uncor*	Uncor*	Uncor*	indir*	indir*	DIRECT*	Uncor*
crude_men	RC		5.260E-03	-3.217E-02	2.751E-02	-9.625E-01	8.652E-04	1.165E-02	3.260E-02
	SE		2.573E-03	1.322E-02	1.323E-02	3.388E-01	2.800E-04	2.870E-03	1.069E-02
model_sex	RC			-2.873E-02	2.659E-02	-7.516E-01	7.776E-04	1.054E-02	2.949E-02

	SE		1.264E-02	1.211E-02	2.998E-01	2.661E-04	2.731E-03	1.040E-02
model_epi	RC			2.498E-02	-6.571E-01	7.364E-04	9.764E-03	2.573E-02
	SE			1.240E-02	3.041E-01	2.652E-04	2.760E-03	1.065E-02
model_dCor	RC			2.706E-02			6.585E-03	
	SE			1.125E-02			3.140E-03	

Upper / lower limits of the 95% confidence interval for a specific RC = RC + / - 1.96 * SE

Independent parameters: the specific LA parameter of men in **crude_men**; the specific LA parameter and sex in **model_sex**; the specific LA parameter, age, sex, WHtR and SBP in **model_epi**; the specific LA parameter, e' velocity, LVEDDI and WHtR in **model_dCor**

* The result of distance and partial distance correlation in Table 4. "Direct" represents an independent correlator while "indir" (indirect) means depending on at least a confounding correlator to be performance correlated. "Uncor" is short for uncorrelation.

LAD = left atrial diameter; LADI = left atrial diameter index; LAEF = left atrial emptying fraction; LAEVI = left atrial emptying volume index; LARVF = left atrial reservoir volume fraction; LASI_m = left atrial stiffness index based on myocardial deformation; LASI_v = left atrial stiffness index based on volume changes; LAVI = left atrial volume index; PALS = peak atrial longitudinal strain; RC = regression coefficient; SE = standard error
